# Comparative structural analyses of the NHL domains from the human E3 ligase TRIM–NHL family

**DOI:** 10.1107/S2052252522008582

**Published:** 2022-09-27

**Authors:** Apirat Chaikuad, Rezart Zhubi, Claudia Tredup, Stefan Knapp

**Affiliations:** a Institute for Pharmaceutical Chemistry, Johann Wolfgang Goethe-University, Max-von-Laue-Strasse 9, D-60438 Frankfurt am Main, Germany; b Structural Genomics Consortium (SGC), Buchmann Institute for Molecular Life Sciences, Johann Wolfgang Goethe-University, Max-von-Laue-Strasse 15, D-60438 Frankfurt am Main, Germany; c German Translational Cancer Network (DKTK), Site Frankfurt/Mainz, German Cancer Research Center (DKFZ), Im Neuenheimer Feld 280, D-69120 Heidelberg, Germany; Chinese Academy of Sciences, China

**Keywords:** TRIM NHLs, NHL domains, TRIM E3 ligases, protein interaction domains, β-propeller protein modules, genetic mutations

## Abstract

Tripartite motif (TRIM) proteins constitute one of the largest subfamilies of the RING-type E3 ubiquitin ligases that play a role in diverse processes from homeostasis and immune response to viral restriction. While TRIM proteins typically harbor an N-terminal RING finger, a B-box and a coiled-coil domain, a high degree of diversity lies in their C termini that contain diverse protein interaction modules, most of which, both structures and their roles in intermolecular interactions, remain unknown. Here, high-resolution crystal structures of the NHL domains of three of the four human TRIM–NHL proteins, namely TRIM2, TRIM3 and TRIM71, are presented. Comparative structural analyses revealed that, despite sharing an evolutionarily conserved six-bladed β-propeller architecture, the low sequence identities resulted in distinct properties of these interaction domains at their putative binding sites for macromolecules. Interestingly, residues lining the binding cavities represent a hotspot for genetic mutations linked to several diseases. Thus, high sequence diversity within the conserved NHL domains might be essential for differentiating binding partners among TRIM–NHL proteins.

## Abstract 2

Family-wide structural analyses of human TRIM NHL domains reveal evolutionary divergence of their β-propeller architecture that might be essential for recruiting diverse interacting partners and for the roles of NHL domains as E3 ligases.

## 1. Introduction

Tripartite motif (TRIM) proteins form one of the largest subclasses of the RING-type E3 ubiquitin ligases, comprising more than 80 members. They commonly harbor a conserved architecture of three N-terminal motifs on their N termini, which include a RING domain, one or two B-box domains and a coiled-coil domain, which are important for E2 binding, protein–protein interaction and oligomerization, respectively (D’Amico *et al.*, 2021 ▸). The presence of these three motifs is a hallmark of this subfamily, thus TRIMs are also referred to as RBCC proteins. However, a high degree of domain variation is observed for the C-terminal regions of TRIMs, which contain various protein modules that typically exert an intermolecular interaction function and thus probably play a significant role in substrate recruitment. Similar to other E3 ligases, TRIMs are key regulators of many biological processes, such as protein quality control and degradation through their involve­ment in ubiquitin–proteasome degradation, autophagy, apoptosis, DNA repair and tumor suppression (D’Amico *et al.*, 2021 ▸; Hatakeyama, 2017 ▸), and, as recently shown, transcription regulation through their RNA-interaction activities (Williams *et al.*, 2019 ▸; Schwamborn *et al.*, 2009 ▸). In addition, emerging evidence has suggested other diverse roles of TRIMs from immune and cell stress responses to viral-entry restriction (Kato *et al.*, 2021 ▸; Caddy *et al.*, 2021 ▸; Stremlau *et al.*, 2004 ▸). These roles overall highlight the importance of TRIMs in homeostasis, and indeed their dysregulation has been linked to diverse disease development (Hatakeyama, 2011 ▸; Meroni, 2020 ▸).

Diversity of the C-terminal protein modules in combination with different numbers of B-box domains lead to the classification of TRIMs into 11 subtypes with an additional ‘unclassified’ group containing members that lack a RING-finger domain (Hatakeyama, 2017 ▸; Williams *et al.*, 2019 ▸; D’Amico *et al.*, 2021 ▸). The SPRY domain, known also as B30.2, is the most common domain, as it appears in more than 40 TRIMs. Other protein modules found at the C termini of TRIMs include, for example, the PHD–Bromo­domain, NHL, COS, filamin and fibronectin type III (FN3), albeit less frequently. These diverse protein domains have different preferences for intermolecular interactions, hence they diversify various biological functions of TRIMs. For example, SPRY domains have been shown to function as a protein–protein interaction module comprising multiple and highly variable binding surfaces for diverse binding partners that have little similarity in topology, share no consensus-sequence motif and play different roles in diverse cellular processes (James *et al.*, 2007 ▸; Kato *et al.*, 2021 ▸). However, PHD–Bromo­domains are more specific protein–protein interaction modules that specifically recognize the acetyl­ated lysine, essentially that of histone, implicating thus the role of this class of TRIMs in epigenetics signaling (Tsai *et al.*, 2010 ▸).

The NHL domain, or so-called NHL repeats named after **n**cl-1, **H**T2A and **l**in-41, forms another protein module found in four TRIMs, including TRIM2, TRIM3, TRIM32 and TRIM71, that constitute the subclass VII. In addition, TRIM56 is another distantly related member due to the presence of an NHL-like domain (Kumari *et al.*, 2018 ▸; Liu *et al.*, 2016 ▸). This motif, which is present in many other proteins, folds into a conserved β-propeller structure; a scaffold that typically mediates interactions with diverse macromolecules including proteins and nucleic acids (Loedige *et al.*, 2015 ▸; Couture *et al.*, 2006 ▸). To date, little is known about human TRIM NHLs and their endogenous interaction partners. However, previous studies on TRIM orthologues established a role of NHL domains as a *bona fide* RNA-binding module (Loedige *et al.*, 2015 ▸; Kumari *et al.*, 2018 ▸; Williams *et al.*, 2019 ▸). This RNA-binding function suggests a role of TRIM–NHL proteins as regulators of gene expression with a link to diverse aspects of RNA metabolisms. In addition, the RNA-binding activity may be important for a role of TRIM56 in suppression of influenza virus RNA synthesis (Liu *et al.*, 2016 ▸).

Although the NHL is evolutionarily conserved, diversity of intermolecular recognitions mediated by the central NHL binding cavities has been proposed, and this property could be linked to plasticity of this protein module that can thus in turn differentiate biological roles of TRIM–NHL proteins (Kumari *et al.*, 2018 ▸). For example, a regulatory role of TRIM71 in controlling expression of genes promoting differentiation has been demonstrated, implicating its involvement in cellular plasticity and reprogramming of differentiated cells into pluripotent cells (Worringer *et al.*, 2014 ▸). TRIM2 NHL has been shown to interact with the motor-protein myosin V, and the function of this E3 ligase has been linked to neuronal activity including neurons and axon growth (Balastik *et al.*, 2008 ▸; Ohkawa *et al.*, 2001 ▸). Diverse biological roles of TRIM32 have been documented ranging from neuronal differentiation, muscle homeostasis and tumor suppression to antiviral infection (Fu *et al.*, 2015 ▸; Schwamborn *et al.*, 2009 ▸; Hillje *et al.*, 2013 ▸; Bawa *et al.*, 2021 ▸). Structural information on the NHL module may provide an underlying molecular basis for distinct solutions to different partners and specific recognitions that may form a key to diverse biological roles of human TRIM–NHL proteins. In this study, we therefore sought to determine the crystal structures and provide comparative analyses of the intrinsic properties of TRIM NHL domains.

## 2. Materials and methods

### 2.1. Recombinant-protein production for TRIM2 and TRIM3 NHLs

The cDNA of the NHL domains of human TRIM2 (aa 466–744; MGC:18215, IMAGE:4156234) and human TRIM3 (aa 466–744; MGC:111679, IMAGE:6108991) were subcloned into pGTVL2, and the proteins were recombinantly expressed as a His_6_-GST fusion in *Escherichia coli*. In brief, the bacteria cultured in TB media were initially grown at 310 K until OD_600_ reached 1.6–1.8. The cultures were then cooled to 291 K, and at an OD_600_ of ∼2.6–2.8, cells were induced with 0.5 m*M* IPTG overnight. The recombinant proteins were initially purified by Ni^2+^-affinity chromatography. The His_6_-GST tag was removed by TEV treatment and the cleaved proteins were separated by passing through Ni^2+^ beads. The proteins were further purified by size-exclusion chromatography using a Superdex s75 column with the buffer containing 20 m*M* HEPES pH 7.5, 200 m*M* NaCl and 0.5 m*M* TCEP.

### 2.2. Recombinant-protein production for TRIM71 NHL

The cDNA of the NHL domain of human TRIM71 (aa 590–868; MGC:190511, IMAGE:100062428) was subcloned into pSUMO-Lic, and the His_6_-Sumo tagged protein was recombinantly expressed in *E. coli*, of which the expression was performed as that described above for TRIM2 and TRIM3 NHLs. The recombinant protein was initially purified by Ni^2+^-affinity chromatography. The expression tag was removed by SENP1 treatment. The cleaved protein was purified by passing through Ni^2+^ beads, and subsequently size-exclusion chromatography using a Superdex s200 column with the buffer containing 20 m*M* HEPES pH 7.5, 200 m*M* NaCl and 0.5 m*M* TCEP.

### 2.3. Crystallization of TRIM NHLs

The recombinant TRIM–NHL domains were concentrated to ∼7.5–10 mg ml^−1^. Crystallization was performed using the sitting-drop vapor-diffusion method at 293 K. The conditions used for growing crystals were (i) for TRIM2, 30%(*w/v*) PEG 5000 MME, 0.2 *M* ammonium sulfate and 0.1 *M* MES, pH 6.5; (ii) for TRIM3, 2.4 *M* ammonium sulfate; and (iii) for TRIM71, 20%(*w/v*) PEG 3350, 0.2 *M* potassium thio­cyanate, 10%(*v/v*) ethyl­ene glycol and 0.1 *M* bis-tris propane, pH 8.5.

### 2.4. Data collection and structure determination

Viable crystals were cryo-protected with mother liquor supplemented with 20%(*v/v*) ethyl­ene glycol for TRIM2 and TRIM71 or 25%(*v/v*) glycerol for TRIM3. Diffraction data were collected at the Swiss Light Source, and were processed and scaled with *XDS* (Kabsch, 2014 ▸) and *AIMLESS* (Evans & Murshudov, 2013 ▸), respectively. Molecular replacement was performed using *Phaser* (McCoy *et al.*, 2021 ▸) and the coordinates of the NHL of *Drosophilla melanogaster* Thin [PDB ID 6d69 (Bawa *et al.*, 2020 ▸)]. The structures were subjected to manual model rebuilding alternated with refinement in *Coot* (Casañal *et al.*, 2020 ▸) and *REFMAC*5 (Kovalevskiy *et al.*, 2018 ▸), respectively. Geometric correctness of the final models was verified by *MolProbity* (Prisant *et al.*, 2020 ▸). The data-collection and refinement statistics are summarized in Table 1 ▸.

## 3. Results and discussion

The NHL domains of TRIM2 and TRIM3 were highly expressed as a fusion protein with an N-terminal His_6_-GST tag in *E. coli*. The same tag was also used for TRIM71, albeit with no success due to a remarkably lower yield and protein instability. An N-terminal His_6_-Sumo tag was instead exploited leading to an improvement of expression levels that enabled successful recombinant-protein production of the TRIM71 NHL domain. We observed that all three recombinant TRIM NHLs without the expression tags behaved as a monomer in gel filtration. With the aim to provide a structural model, we attempted crystallization and gratifyingly obtained the crystals of all three proteins. Crystals of TRIM2 NHL were obtained within 1–2 d, while TRIM3 crystals grew within one week and TRIM71 crystals formed after approximately one month. All crystals showed good X-ray diffraction quality, enabling high-resolution structure determination. For TRIM2, the structure was refined to a high resolution of 1.45 Å, and the crystals belonged to the monoclinic *P*2_1_ space group with four molecules in the asymmetric unit. The TRIM3 NHL structure was determined at 1.7 Å resolution from the tetragonal crystals that contained a single molecule in the asymmetric unit, whereas the monoclinic crystals of TRIM71 NHL diffracted to 2.2 Å resolution had an asymmetric unit consisting of two protein molecules.

**Figure 1:**
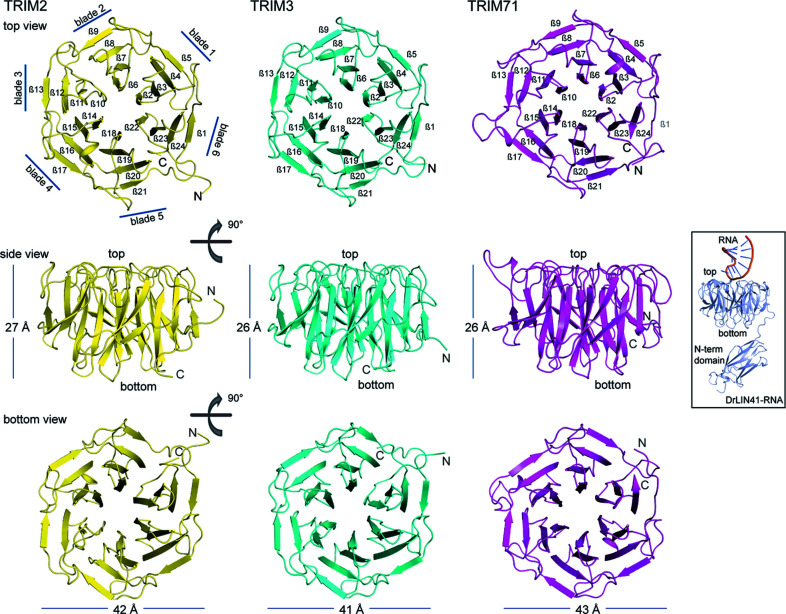
An overview of the TRIM2, TRIM3 and TRIM71 NHL domain structures. The cartoon representations show the overall structures of the TRIM NHL β propellers with top, side and bottom views. The inset in the middle is the structure of zebrafish *Dr*LIN41 filamin–NHL domain in complex with an RNA (PDB ID 6fpt), displayed in the same orientation as that used in the middle panel to highlight the top and bottom of the binding cavities of the TRIM–NHL domains.

All three TRIM NHLs shared a highly similar tertiary structure by adopting the canonical β-propeller topology, which was previously described for these protein modules in the homologues *Danio rerio* Lin41 (*Dr*LIN41) [PDB ID 6fpt (Kumari *et al.*, 2018 ▸)], *D. melanogaster* Thin (*Dm*Thin) (Bawa *et al.*, 2020 ▸) and *D. melanogaster* Brain tumor (*Dm*Brat) (Edwards *et al.*, 2003 ▸). In brief, the TRIM–NHL propellers were built from six β-sheet blades, each having an identical construction consisting of four β strands (Fig. 1 ▸). The N-terminal starting point and the C-terminal end of the propeller were located at a similar position and were a part of the sixth β sheet. Such highly similar architecture resulted in similar dimensions for all three NHL domains with a diameter of ∼42 Å and a thickness of ∼26 Å.

**Figure 2:**
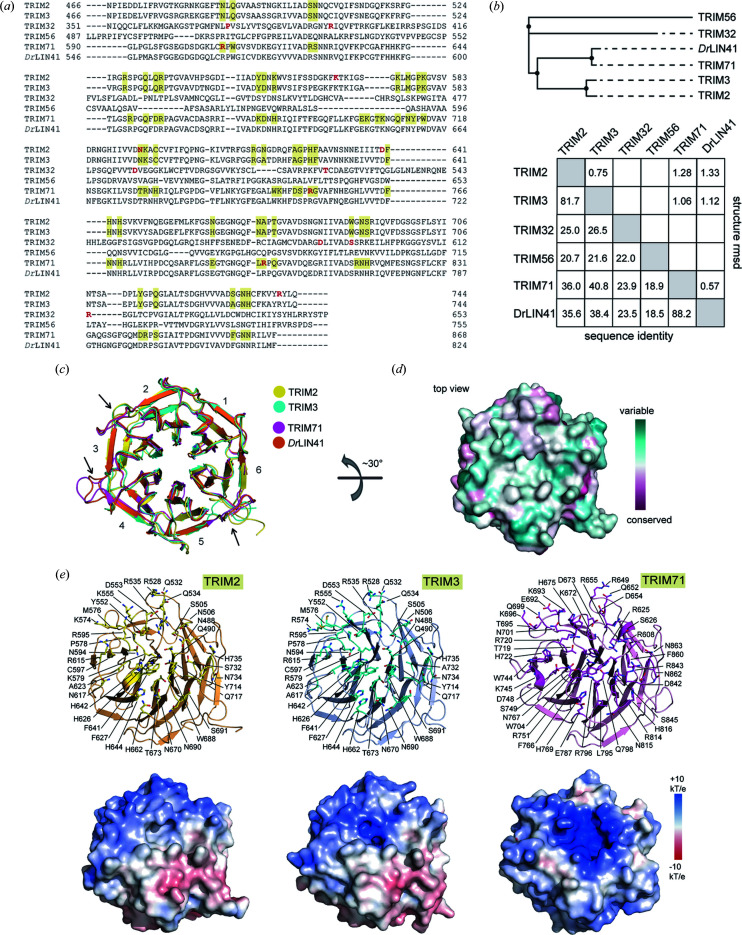
Comparative analyses of the TRIM NHLs. (*a*) Sequence alignment of the NHL motifs from all four human TRIMs within subfamily VII, the NHL-like domain of TRIM56 and the NHL domain of the homologue *Dr*LIN41. The yellow boxes highlight the amino acids in the vicinity of the central groove of the top opening surface, while amino acids in red are the genetic mutations linked to diseases (see also Fig. 3 ▸). (*b*) A sequence-based phylogenetic tree for the NHL and NHL-like domains of human TRIMs and their homologue *Dr*LIN41. Below are the sequence identities and r.m.s.d. calculated from structural comparison between the pairs. (*c*) Superimposition of the TRIM–NHL structures reveals highly conserved topology of the β-propeller architectures, yet slight differences are observed for some connecting loops (arrows). (*d*) Sequence conservation at the top opening among human TRIM NHLs calculated using *ConSurf* (Ashkenazy *et al.*, 2016 ▸). (*e*) Mapping of amino acid compositions (top) and electrostatic potentials (bottom) of the central grooves at the top surface of TRIM2, TRIM3 and TRIM71 NHLs.

The high structural homology was unexpected considering the low level of sequence similarity of only 19–41% among the NHL motifs of the four members of the TRIM–NHL family (TRIM2, TRIM3, TRIM32 and TRIM71) and the NHL-like domain of TRIM56 [Figs. 2 ▸(*a*) and 2 ▸(*b*)]. Nonetheless, an exception was noted when comparing TRIM2 and TRIM3 that were most similar with ∼82% sequence identity. In contrast, high similarity was observed when comparing TRIM family paralogues from different species, exemplified by, for instance, an 88% identity between human TRIM71 and zebrafish *Dr*LIN41 [Fig. 2 ▸(*b*)]. This suggests that, barring the TRIM2–TRIM3 pair, each NHL domain of human TRIMs might emerge from different ancestors and paralogues, and remain evolutionarily conserved based on phylogenetic relationships [Fig. 2 ▸(*b*)].

At a three-dimensional structural level, despite the high sequence differences, the β-propeller architectures of the TRIM2, TRIM3 and TRIM71 NHLs remained highly conserved, revealed by highly superimposable structures with pair-wise r.m.s.d. values of 0.75–1.33 Å [Fig. 2 ▸(*b*)]. Nonetheless, some structural variations were still observed, and these were located mainly at the rim of the binding pockets. Notable differences included the lengths and conformations of the blade-connecting loops, especially those that linked blades 2 and 3, 3 and 4, and 5 and 6 [Fig. 2 ▸(*c*)], of which some degrees of variation were also seen among the NHL domains of homologues *Dr*LIN41, *Dm*Brat, *Dm*Mei-P26 (Salerno-Kochan *et al.*, 2022 ▸) and *Dm*Thin (Bawa *et al.*, 2020 ▸) (see Fig. S1 of the supporting information). However, based on the RNA-complexed structures of *Dr*LIN41 and *Dm*Brat, the parts of these loops with structural differences did not directly involve the binding of the substrate (Fig. S1). We speculated therefore that such conformational alterations may play a role in the maintenance of intrinsic structural integrity rather than directly participating in intermolecular inter­actions.

Further comparative sequence conservation analyses indeed confirmed high diversity around these blade-connecting loops as well as at the top opening with distinct amino acid compositions that constitute the binding site [Figs. 2 ▸(*a*), 2 ▸(*d*) and 2 ▸(*e*)]. Such differences resulted in an overall high diversity in shape and electrostatic properties of the putative intermolecular interface [Fig. 2 ▸(*e*)]. A less polar shallow groove surrounded by mixed positively and negatively charged patches was observed for the binding sites of TRIM2 and TRIM3, whereas a strong positively charged surface with a deeper central hole unveiled a unique characteristic of TRIM71 [Fig. 2 ▸(*e*)]. It is tempting to speculate that such distinct properties were probably constructed by coevolution of diverse NHL binding partners. For example, the highly positively charged interface with a deep central groove suggests potentially a similar function of TRIM71 NHL to that of the homologue *Dr*LIN41 as an interacting protein module that accepts RNA substrates harboring a stem-loop motif (Kumari *et al.*, 2018 ▸) (Fig. S2). In contrast, the rather shallow flat surface of the TRIM2/3 NHLs resembles that of their homologue *Dm*Brat, which has been shown to recognize a linear RNA (Loedige *et al.*, 2015 ▸). However, comparative analyses suggest that similar binding of an RNA observed previously in *Dm*Brat would be unlikely in TRIM2/3 due to the lack of a central channel as well as low sequence conservation within the pockets (Fig. S2).

All protein domains of TRIMs are known to serve as essential intermolecular interaction modules required for E3 ligase activity. This includes a RING domain for E2 binding and B-box and coiled-coil domains for intermolecular interactions and/or oligomerization (D’Amico *et al.*, 2021 ▸). The C-terminal domains, such as the PHD–Bromo­domain (Tsai *et al.*, 2010 ▸), SPRY (James *et al.*, 2007 ▸) and NHL (Kumari *et al.*, 2018 ▸; Edwards *et al.*, 2003 ▸), have been reported as recognizable protein interacting modules, which may likely be utilized for substrate recruitment. Dysfunctions of these domains potentially lead to an impairment of TRIM E3 ligase functions and deregulation of ubiquitin-mediated signaling, which could form a cause of multiple diseases including neurological disorders and cancers, as well as many rare diseases (Meroni, 2020 ▸; Hatakeyama, 2011 ▸; Balastik *et al.*, 2008 ▸). In line with this, genetic studies have unveiled a number of mutations in the NHL motifs of TRIMs with a link to diverse pathological outcomes, in particular, diverse neurological disorders. This includes a link of the mutations in TRIM2 NHL to Charcot-Marie-Tooth disease (CMT), congenital bilateral vocal-cord paralysis (BVCP) and axonal neuropathy (Pehlivan *et al.*, 2015 ▸; Ylikallio *et al.*, 2013 ▸; Magri *et al.*, 2020 ▸; van Diepen *et al.*, 2005 ▸). In addition, the mutations in TRIM71 NHL have been associated with congenital hydro­cephalus (Welte *et al.*, 2019 ▸; Furey *et al.*, 2018 ▸), while those in TRIM32 NHL have been linked to myopathy such as limb-girdle muscular dystrophy type 2H (LGMD2H) and sarcotubular myopathy (STM) (Schoser *et al.*, 2005 ▸; Frosk *et al.*, 2002 ▸, 2005 ▸; Kudryashova *et al.*, 2011 ▸; Yu *et al.*, 2017 ▸; Saccone *et al.*, 2008 ▸; Panicucci *et al.*, 2019 ▸; Neri *et al.*, 2013 ▸).

**Figure 3:**
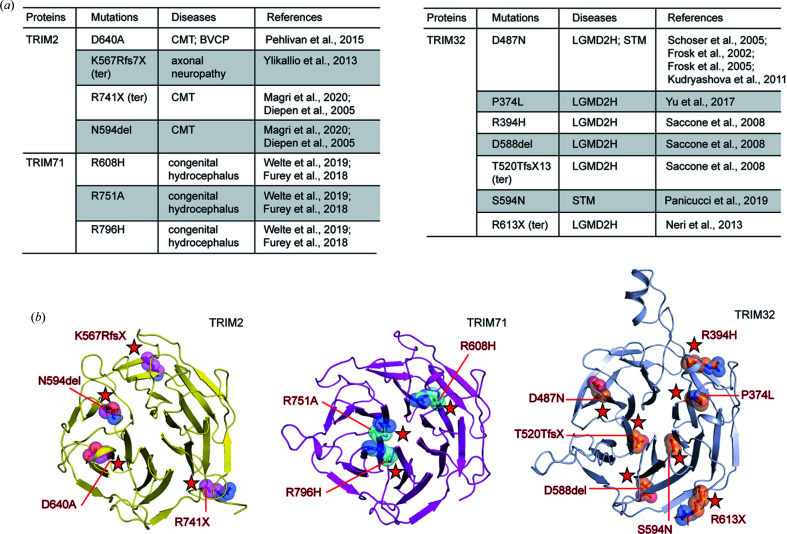
Disease-linked mutations in TRIM NHLs. (*a*) A list of reported genetic mutations linked to diverse diseases. (*b*) Mapping of the disease-associated mutations on the crystal structures of TRIM2 and TRIM71 NHLs and the *AlphaFold* model of TRIM32 NHL.

Strikingly, most of the genetic mutations in the NHL-containing TRIMs are located within the NHL domain, and we summarize these known mutations in Fig. 3 ▸(*a*). Several types of mutations were identified, including amino acid substitutions and deletions as well as nonsense mutations that lead to an early transcription termination, hence a loss of the NHL protein domain. We used the crystal structures of TRIM2 and TRIM71 as well as the *AlphaFold* model of TRIM32 (Jumper *et al.*, 2021 ▸) to map the locations of the mutations onto the NHL domains. Although these disease-linked mutations are distributed throughout the propeller structure, the putative intermolecular interaction surface interestingly forms a hotspot [Fig. 3 ▸(*b*)]. For TRIM2 NHL, two nonsense mutations were reported. A frameshift within blade 2 (K567Rfs7X) undoubtedly leads to a loss of the NHL domain, whereas the other nonsense mutation (R741X) results in the loss of four amino acids at the C terminus on the bottom surface. The other mutations include a substitution, D640A, and a deletion, N594del, both of which are located in the proximity of the central groove on the top surface, thus they could affect directly the integrity of the intermolecular interface. Such similar effect would also likely be anticipated for all three amino acid substitutions in TRIM71 [Fig. 3 ▸(*b*)]. These disease-linked mutations involving the changes of three positively charged arginine residues at the top surface peripheral to the central channel to a shorter hydro­phobic alanine or histidine would alter the characteristics of strong positively charged electrostatic potentials of the binding interface, probably built for the interaction with the phosphate backbone of nucleic acids as seen in the *Dr*LIN41 homologue (Kumari *et al.*, 2018 ▸).

Among the TRIM–NHL members, TRIM32 has the highest number of reported genetic mutations, all of which have been associated with rare muscle disorders. Although we were not successful in determining the crystal structure of TRIM32 NHL, we used an *AlphaFold* model to map the locations of the mutations. We found that, consistent with TRIM2 and TRIM71, the reported mutations are located in the vicinity of the rim surface of the central cavity [Fig. 3 ▸(*b*)]. Sequence alignment showed similarly that these mutated residues in TRIM32 clustered within the groups of amino acids that were found to line the binding interfaces of TRIM2, TRIM3 and TRIM71 [Fig. 2 ▸(*a*)]. Analyses of the mutations suggested that the nonsense mutations in blade 4 (T520TfsX) and blade 5 (R613X) would result in a loss of the NHL module, whereas substitutions and deletion leading to the changes both in charge properties such as R394H, D487N and D588del and in sizes such as P374L and S594N could alter the physical properties that may affect the function of this protein domain.

## 4. Conclusions

The NHL motif is an evolutionarily conserved protein domain that has been found in many proteins. NHL domains are also present at the C termini of four human E3 ligase TRIMs, including TRIM2, TRIM3, TRIM32 and TRIM71, as well as TRIM56 that harbors an NHL-like domain. This protein module folds into a β-propeller architecture that mediates intermolecular interaction, with a function as a *bona fide* RNA-binding module established for the homologues from various eukaryotes (Kumari *et al.*, 2018 ▸; Loedige *et al.*, 2015 ▸). We have presented here the crystal structures of the NHLs from TRIM2, TRIM3 and TRIM71, providing structural insights for this domain in human TRIM–NHL proteins. Despite sharing a highly conserved three-dimensional topology, our structural models revealed significant differences in the central NHL binding pockets, comprising a high degree of variation in shape, amino acid compositions and electrostatic potentials. The highly diverse rim surface of the binding cavity probably serves as a binding site for highly diverse interaction partners. We found that this region was also a hotspot of genetic mutations linked to the development of diseases including several neurological and muscle dis­orders. Overall, our structural information highlights evolutionary divergence that differentiates intrinsic properties and potentially recognition functions of this conserved protein domain, diversifying the biological functions of TRIM–NHL proteins. In addition, these structures may serve as a template for further study to identify the interaction partners as well as the functions of these TRIM NHLs and potentially the development of small molecule binders that, in a similar manner to other β-propeller protein modules (Wei *et al.*, 2021 ▸), might find applications in the development of protein-targeting chimeras and molecular glues.

## Supplementary Material

Supporting information. DOI: 10.1107/S2052252522008582/lz5059sup1.pdf

PDB reference: NHL domain of TRIM71, 7qrx

PDB reference: NHL domain of TRIM3, 7qrw

PDB reference: NHL domain of TRIM2 (full C terminal), 7qrv

## Figures and Tables

**Table 1:** Data-collection and refinement statistics

Complex	TRIM2 NHL	TRIM3 NHL	TRIM71 NHL
PDB accession code	7qrv	7qrw	7qrx
			
Data collection			
Resolution (Å)†	45.43–1.45 (1.49–1.45)	47.58–1.70 (1.74–1.70)	45.71–2.20 (2.28–2.20)
Space group	*P*2_1_	*P*4_1_2_1_2	*P*2_1_
Cell dimensions	*a* = 68.7 Å, *b* = 89.2 Å, *c* = 86.7 Å, α, γ = 90.0°, β = 107.0°	*a* = *b* = 58.9 Å, *c* = 161.2 Å, α, β, γ = 90.0°	*a* = 34.7 Å, *b* = 78.7 Å, *c* = 92.9 Å, α, γ = 90.0°, β = 100.1°
No. unique reflections†	175817 (12684)	31138 (2171)	25069 (2450)
Completeness (%)†	99.5 (97.4)	97.1 (94.5)	99.9 (99.9)
〈*I*/σ(*I*)〉†	16.8 (3.8)	24.6 (3.8)	7.6 (2.1)
*R*_merge_ †	0.059 (0.411)	0.058 (0.587)	0.133 (0.817)
CC_1/2_	0.999 (0.922)	0.999 (0.940)	0.998 (0.861)
Redundancy†	6.8 (6.7)	16.0 (17.3)	6.9 (7.1)
			
Refinement			
No. atoms in refinement (P/O)‡	8620/1377	2152/162	4347/218
*B* factor (P/O) (Å^2^)‡	16/29	37/44	38/39
*R*_fact_ (%)	15.2	18.2	23.2
*R*_free_ (%)	17.8	21.5	27.2
R.m.s.d. bond (Å)	0.016	0.016	0.009
R.m.s.d. angle (°)	1.6	1.5	1.1
			
Ramachandran analysis			
Favored (%)	95.66	94.53	95.13
Allowed (%)	3.98	4.74	4.87
Outliers (%)	0.36	0.73	0

† Values in brackets show the statistics for the highest resolution shells.

‡ P/O indicates protein and others (water and solvent molecules), respectively.
