# Supplementary material for: Comparative structural analyses of the NHL domains from the human E3 ligase TRIM–NHL family
Source: IUCrJ. 2022 Sep 27;9(Pt 6):720–7. doi: 10.1107/S2052252522008582 (PMC9634614; doi:10.1107/S2052252522008582)
Supplement: Supplementary file 1 [file m-09-00720-sup1.pdf]

IUCrJ

Volume 9 (2022)

Supporting information for article:

Comparative structural analyses of the NHL domains from the human E3 ligase TRIM-NHL family

Apirat Chaikuad, Rezart Zhubi, Claudia Tredup and Stefan Knapp

Supplementary information

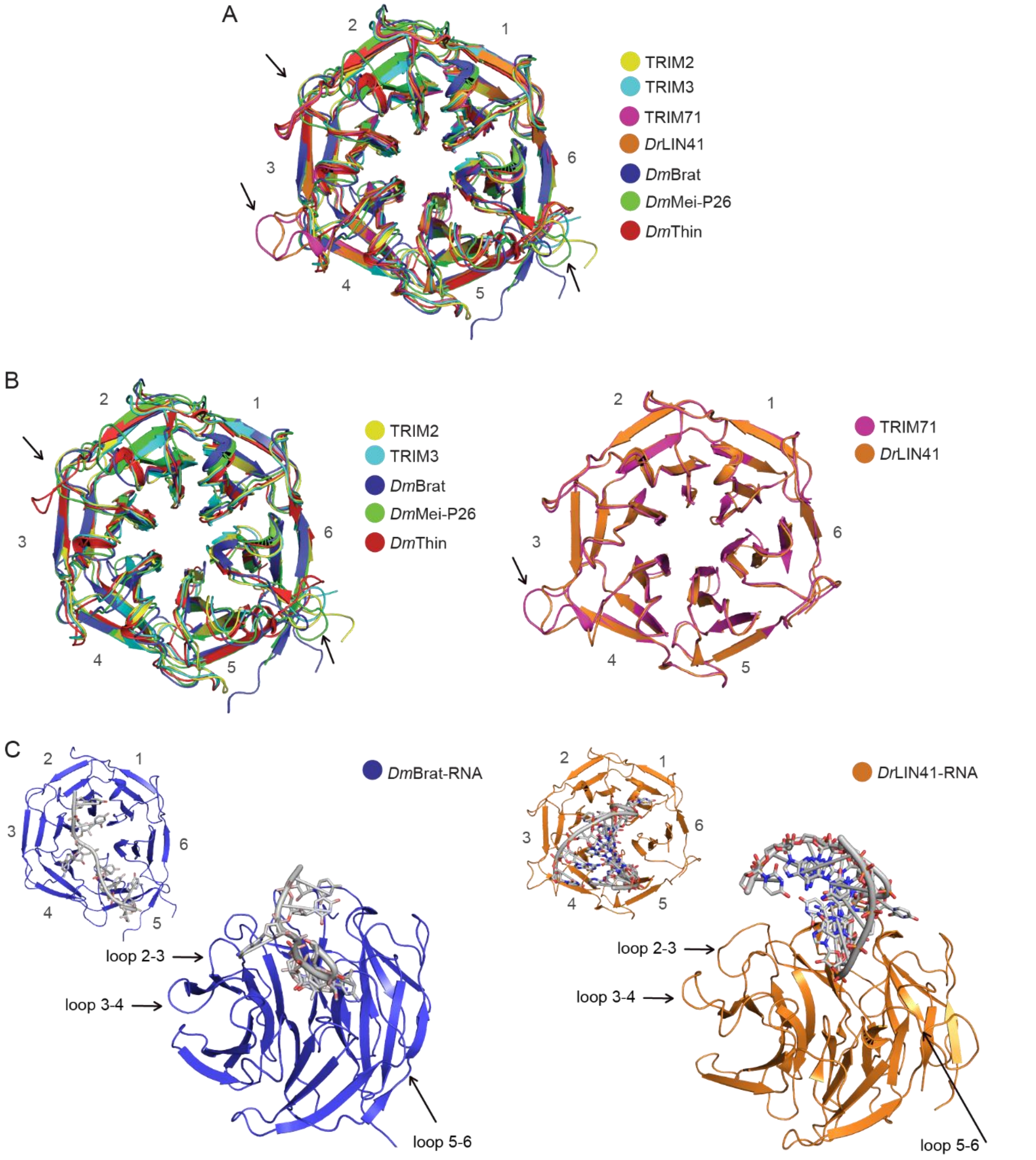

Supplementary Figure S1. Structural comparison of TRIM2/3/71 and their homologues. A, B) Superimposition of the structures of the NHL domains of TRIM2/3/71, *Dr*Lin41 (pdb id: 6fql), *Dm*Brat (pdb id: 4zlr), *Dm*Mei-P26 (pdb id: 7nyq) and *Dm*Thin (pdb id: 6d69). Arrows highlight the interblade loops that exhibit conformational variation. C) The RNA-complexed *Dr*Lin41 (pdb id: 6fql) and *Dm*Brat (pdb id: 4zlr) showing the locations of interblade loop 2-3, 3-4 and 5-6 peripheral to the binding pockets.

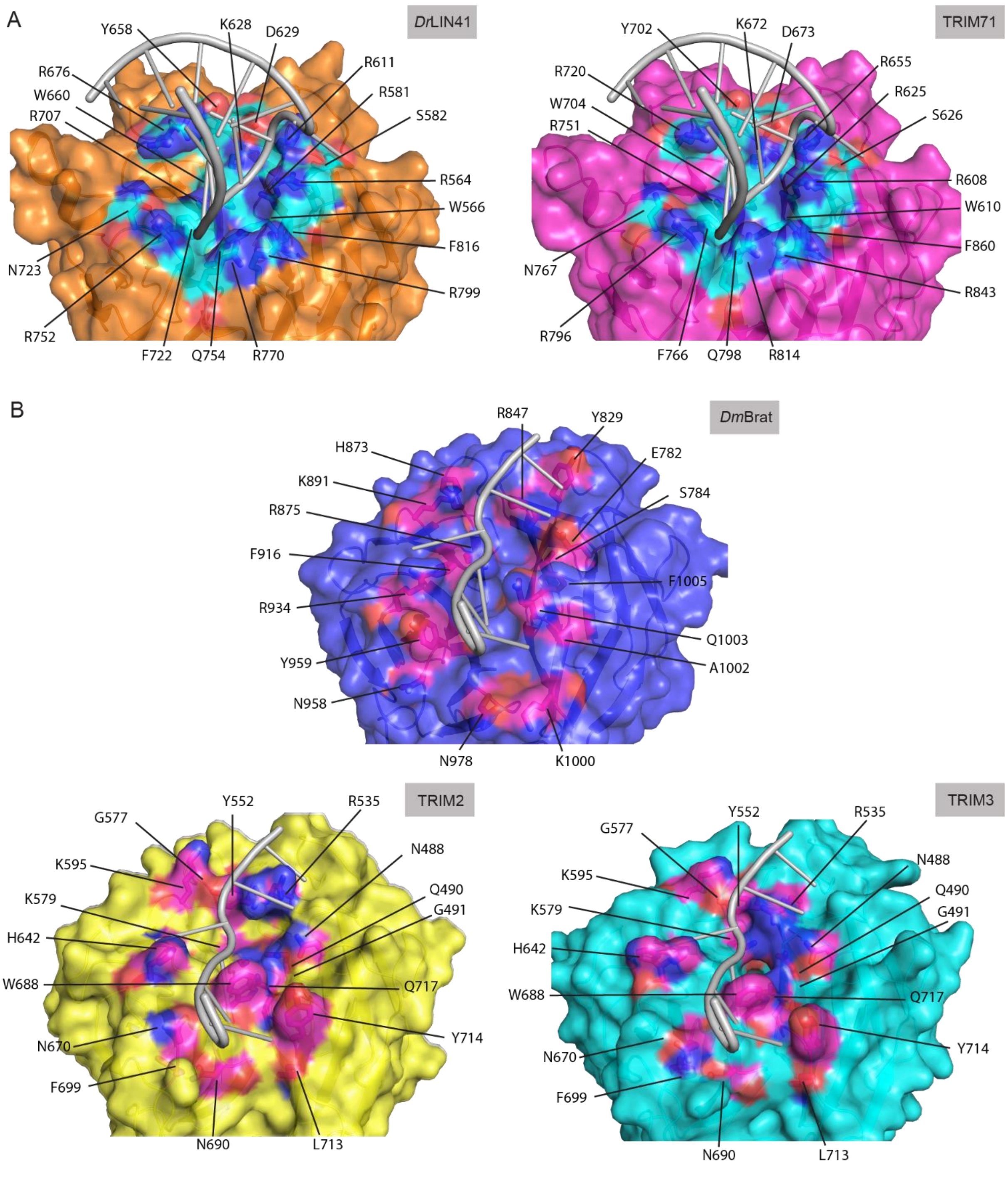

Supplementary Figure S2. Comparison of the binding pockets of TRIM2/3/71 and their homologues. Surface representation showing the binding pockets of TRIM71 and its homologue *Dr*Lin41 (top, A) and TRIM2/3 and their homologue *Dm*Brat (bottom, B). The bound RNA molecules from *Dr*Lin41 (pdb id: 6fql) and *Dm*Brat (pdb id: 4zlr) structures are shown in grey cartoon representation. Residues in vicinity of the binding site are highlighted and labelled.
